# The *Sinorhizobium meliloti* Nitrogen Stress Response Changes Radically in the Face of Concurrent Phosphate Stress

**DOI:** 10.3389/fmicb.2022.800146

**Published:** 2022-01-27

**Authors:** Kelly L. Hagberg, Jason P. Price, Svetlana N. Yurgel, Michael L. Kahn

**Affiliations:** ^1^School of Molecular Biosciences, Institute of Biological Chemistry, Washington State University, Pullman, WA, United States; ^2^Department of Plant, Food and Environmental Sciences, Dalhousie University, Truro, NS, Canada

**Keywords:** *Sinorhizobium*, nitrogen stress, phosphate stress, stress responses, P_*II*_ proteins

## Abstract

Expression of hundreds of *S. meliloti* genes changed more than two-fold in response to either nitrogen or phosphate limitation. When these two stresses were applied together, stress responsive gene expression shifted dramatically. In particular, the nitrogen stress response in the presence of phosphate stress had only 30 of about 350 genes in common with the 280 genes that responded to nitrogen stress with adequate phosphate. Expression of sRNAs was also altered in response to these stresses. 82% of genes that responded to nitrogen stress also responded to phosphate stress, including 20 sRNAs. A subset of these sRNAs is known to be chaperoned by the RNA binding protein, Hfq. Hfq had previously been shown to influence about a third of the genes that responded to both nitrogen and phosphate stresses. Phosphate limitation influenced changes in gene expression more than nitrogen limitation and, when both stresses were present, phosphate stress sometimes reversed the direction of some of the changes induced by nitrogen stress. These nutrient stress responses are therefore context dependent.

## Introduction

Soils vary substantially in characteristics that influence bacterial growth and survival, such as nutrient or water availability, salt content, pH, and the abundance of competing and predatory organisms (Zahran, 1999; Vitousek et al., 2010; Fanin et al., 2016). Bacteria have developed pathways to cope with environmental changes and can respond to various growing conditions in ways believed to optimize their success under stressful conditions. Low levels of usable nitrogen and phosphate are the most commonly limiting nutrients (Vitousek et al., 2010; Fanin et al., 2016) and the ability to cope with nitrogen or phosphate starvation is important. Rhizobia are best known for their nitrogen-fixing symbiotic relationships with legumes in which the bacteria convert atmospheric dinitrogen into ammonium, which can then be assimilated by the legumes and support plant growth. This chemically available nitrogen is an important input in natural and agricultural ecosystems (Bohlool et al., 1992; Mylona et al., 1995). Free-living bacteria initiate these symbioses through infection of roots, making persistence of rhizobia in diverse soils an important precondition for success of the relationships. Since several stresses can occur simultaneously in natural ecosystems, understanding how stress response mechanisms affect each other is important in understanding bacterial stress adaptation.

Historically, the nitrogen stress response (NSR) and the phosphate stress response (PSR) have been characterized separately by limiting only the designated nutrient in an otherwise complete growth medium. The general concept underlying these studies is that gene expression changes observed in response to the nutrient stress help the organism cope with low levels of the limiting nutrient. The experimental design of single nutrient limitation also helps to define the regulatory circuits that mediate the stress response. Crosstalk between stress responses has been seen when they share common regulatory elements such as RNA polymerase sigma subunits or, in the case of two-component regulatory systems, when the histidine kinase sensor proteins are able to partially modify non-cognate response regulators (Mike et al., 2014; Choudhary et al., 2020).

*Sinorhizobium meliloti* is a symbiont of alfalfa and other *Medicago* species. Its nitrogen and phosphate stress responses have been studied individually and key proteins and genes have been characterized for each pathway (Al-Niemi et al., 1997; Voegele et al., 1997; Patriarca et al., 2002; Krol and Becker, 2004; Yurgel et al., 2012). The primary sensor for the NSR is GlnD, a uridylyltransferase that modifies the P_*II*_ proteins GlnB and GlnK in response to nitrogen availability (Merrick and Edwards, 1995; Ikeda et al., 1996; Jiang et al., 2012). P_*II*_ proteins are common among bacteria and their modification state affects how they regulate several NSR components, including phosphorylation of the NtrB/NtrC two-component regulatory system. NtrC-P works with the RpoN sigma factor to alter expression of genes involved in enhancing nitrogen uptake and efficiently utilizing available nitrogen (Merrick and Edwards, 1995; Arcondeguy et al., 2001; Leigh and Dodsworth, 2007). The activity of glutamine synthetase (GS), which can assimilate low levels of ammonium into amino acids by coupling assimilation to ATP hydrolysis, is well characterized and is often used as a marker of the activation state of the NSR. GS is abundant and highly active during nitrogen limitation and has lower abundance and specific activity when nitrogen levels are sufficient (Yurgel et al., 2010). *S. meliloti* has two major GS enzymes: GSI, which is encoded by *glnA* and regulated primarily through posttranslational modification (Atkinson and Ninfa, 1998; Yurgel et al., 2010), and GSII, which is encoded by *glnII* and primarily regulated through transcriptional control by the action of the NtrB/NtrC two-component regulatory system (Arcondéguy et al., 1997).

The *S. meliloti* phosphate stress response (PSR) is regulated by the PhoR/PhoB two-component regulatory system. When phosphate is limiting, the phosphorylated form of the PhoB response regulator (PhoB-P) works with the RpoD sigma factor to alter target gene expression with the apparent goal of enhancing uptake and efficient use of available phosphate while downregulating less critical processes that consume phosphate (Makino et al., 1993; Al-Niemi et al., 1997). Alkaline phosphatase (AP), which releases phosphate from phosphate esters, is often used as an indicator of PSR activity, similar to using GS as an index of the NSR. In *S. meliloti*, a major AP is encoded by *phoX*, and its expression depends on PhoB-P binding to the *phoX* promoter region (Zaheer et al., 2009). *S. meliloti* has several phosphate transport systems (Pst, Pho, and Pit) that are regulated by the PSR and differ in their affinity and capacity for phosphate uptake (Voegele et al., 1997; Yuan et al., 2006a).

Adding nitrogen or phosphate can improve growth of microorganisms within an environment and can change the microbiome population dynamics to favor either bacteria or fungi (Vitousek et al., 2010; Fanin et al., 2016). Adding both N and P has a synergistic effect on autotrophic microorganism growth (Elser et al., 2007), supporting the idea that stress responses are coordinated and that the responses to multiple nutrient limitations are integrated. This idea contrasts with a model in which the stress responses are independent pathways that are activated in response to single nutrients. In the latter model, a set of stress response genes would be regulated by the primary nutrient regardless of other stresses, but a handful of additional genes might be affected by imposing a second stress.

Recent studies in *S. meliloti* identified interactions between the NSR and PSR (Yurgel et al., 2013; Hagberg et al., 2016) as well as environmental signals affecting two-component systems including PhoB (Choudhary et al., 2020). A microarray experiment identifying nitrogen responsive genes revealed that deleting the *glnB* and *glnK* genes, which encode the P_*II*_ proteins, influenced expression of many more genes than are controlled by the NSR, including genes known to respond to phosphate limitation (Yurgel et al., 2013). Previous data also show that GS is regulated by phosphate in addition to nitrogen (Hagberg et al., 2016). In contrast, AP is regulated by phosphate, but nitrogen availability did not substantially influence AP activity or expression (Hagberg et al., 2016). In proteobacteria like *S. meliloti*, the cellular response to nitrogen is integrated with carbon metabolism and storage as well. The phosphotransferase system (PTS) regulates nitrogen and carbon pools to maintain balance; for example, when nitrogen is limiting, the PTS system will work with the NSR to enhance nitrogen uptake and metabolism while also altering carbon metabolism and storage (Sánchez-Cañizares et al., 2020). sRNAs have recently been shown to play a role in fine-tuning the balance of the nitrogen and carbon pools (Prasse and Schmitz, 2018).

The *E. coli* nitrogen and phosphate stress response pathways and regulatory proteins are very similar to the *S. meliloti* pathways described above. PhoB is stimulated by phosphate stress, but there is also stimulation by cell envelope stress which highlights the cross-regulation of the well-characterized PhoB regulatory protein (Choudhary et al., 2020). Recently, Sastry et al. (2019) described nitrogen and phosphate “imodulons” in *E. coli* as groups of genes whose expression moves in similar patterns. They speculated that bacteria respond to nutrient stresses in ways that integrate two major drivers of bacterial behavior: greed and fear. “Greed” describes the bacterial motivation to grow and includes changing expression of genes to favor reproduction in response to limited amounts of available nutrient. “Fear” describes the bacterial motivation to survive and includes changing expression of genes to allocate resources to essential and protective functions in order to avoid cell death. So, for example, limiting the amount of ammonia available to the culture may induce genes that allow it to use alternative forms of nitrogen and, if successful, would allow the culture to resume growing using this alternate nutrient. Activating a generalized shutdown of related metabolic pathways, like the stringent response induced by amino acid starvation, might be considered a “fear” response that blocks immediate resource use in favor of a more conservative allocation.

The cross-regulation observed in *E. coli* and the response of GS expression and activity to both nitrogen and phosphate in *S. meliloti* led us to ask whether other genes, NSR-related or not, also respond to both nutrients. In addition, we were interested in how *S. meliloti* prioritized its responses to nitrogen and phosphate availability and to see if the speculated fear vs. greed priorities change as nutrient stresses stack on one another. An RNA-Seq experiment was designed to explore global changes in mRNA and sRNA abundance in response to limited nitrogen, limited phosphate, or both.

The results below demonstrate that, at least for the nitrogen stress response, the set of genes that responded to nitrogen stress in the presence of high phosphate was very different from the set that responded in a low phosphate environment, and that only a small fraction of the large number of “nitrogen-responsive genes” found could be characterized as nitrogen-responsive under both phosphate conditions. A similar pattern was observed with phosphate responsive genes in the presence or absence of nitrogen stress, but the overlap of the two groups of phosphate-responsive genes was larger. We briefly discuss genes that were both nitrogen and phosphate responsive, for which responding to phosphate limitation appeared to have higher priority than responding to nitrogen limitation. We speculate about the existence of two different stress responses to a single nutrient under the different stress conditions.

## Materials and Methods

### Bacterial Strains and Media

*Sinorhizobium meliloti* Rm1021 *pstC*+ and Rm1021*glnBK pstC*+ strains (Hagberg et al., 2016) were used. Cultures were grown at 30°C in a shaking incubator in Minimal MOPS media with 1% mannitol as the carbon source, 0.4% NH_4_^+^ (high N) or 0.04% glutamate (low N) as nitrogen sources and 2 mM KH_2_PO_4_ (high P) or 50 μM KH_2_PO_4_ (low P) as phosphate sources. Induction of NSR and PSR by the low concentrations of these nutrients has been previously studied (Al-Niemi et al., 1997; Hagberg et al., 2016).

### RNA Isolation, rRNA Depletion, cDNA Library Formation, and RNA-Seq

Cultures were grown in supplemented Min MOPS (Hagberg et al., 2016) medium to an A_600_ of 0.500 ± 0.026. Cells were harvested by centrifugation at 20,000 *g* for 10 min at 4°C. Cell pellets from 36 mL of culture were suspended in 200 μL lysis buffer [1× phosphate buffered saline (137 mM NaCl, 2.7 mM KCl, 7.75 mM Na_2_HPO_4_, 1.47 mM KH_2_PO_4_), 1 mM EDTA, and 0.1% Triton-X]. Cells were sonicated for 10 sec, placed on ice for 1 min, sonicated for 15 s, iced for one min, and sonicated for 15 s. Lysates were spun at 20,000 *g* for 15 min at 4°C and the supernatant was added to 1 mL TRIzol reagent (Thermo Fisher Scientific, Waltham, MA, United States). Standard procedures were followed for phase separation, RNA precipitation and RNA washing with TRIzol. RNA was further purified using the QIAGEN RNeasy kit. RNA was processed by using the Illumina TruSeq Stranded Total RNA kit following standard procedures at half reaction strength per sample to deplete the samples of rRNA and generate and enrich cDNA libraries with indexed adaptors. The cDNA libraries were validated by DNA fragment analysis. Standard Qubit 2.0 procedures were used to measure library sample concentrations which were normalized by qPCR prior to pooling them. Samples were sequenced with 50 bp, single end read conditions using an Illumina 2500 at the Washington State University (WSU) Genomics core facility in Spokane, WA, United States.

### Data Analysis

FASTQ files from the RNA sequencing were analyzed using the Tuxedo Suite of programs. Reads were mapped with Tophat (Trapnell et al., 2012) to the closely related *S. meliloti* 2011 genome, which has a more complete annotation than the *S. meliloti* 1021 genome and includes non-coding sRNAs, and was then analyzed further with Cuffdiff, which allowed comparison of samples to determine significant expression changes between medium conditions (Trapnell et al., 2013). Mapped reads were analyzed with the R bioinformatics software WGCNA for information about weighted co-expression of genes (Langfelder and Horvath, 2008). The weighted co-expression data was visualized using Cytoscape to generate a co-expression network map (Shannon et al., 2003). The heatmap3 R package was used to generate a heatmap of significant differentially expressed genes (Zhao et al., 2014).

### Experimental Design and Analytical Comparisons

**FIGURE 1:**
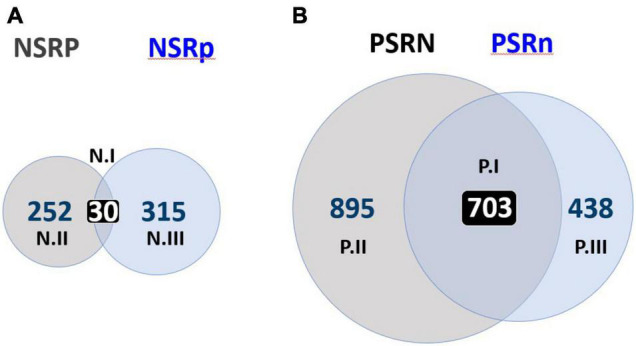
Overview of gene expression changes under Nitrogen and Phosphate stress. Venn diagrams of genes that respond to nitrogen or phosphate stress in the presence or absence of the other stress. Stress responses are labeled by indicating primary nutrient comparison (i.e., NSR, nitrogen stress response or PSR, phosphate stress response) and then adding an upper- or lower-case letter for the other nutrient involved (i.e., NSRP for nitrogen stress response under high phosphate, NSRp for nitrogen stress response under low phosphate). **(A)** Venn diagram showing genes where mRNA abundance changed two-fold (*p* ≤ 0.01) when comparing ammonia-grown cultures to glutamate–grown cultures under sufficient phosphate (gray = NSRP) and under limiting phosphate (blue = NSRp). **(B)** Genes where mRNA abundance changed two-fold (*p* ≤ 0.01) when comparing 2 mM phosphate-grown cultures to 50 μM phosphate-grown cultures with ammonium as nitrogen source (gray = PSRN) or glutamate as the nitrogen source (blue = PSRn). Each section of the Venn diagram is labeled with the cluster name as defined in Table 2 and the number of genes included in that cluster.

To test the interactions between bacterial nitrogen and phosphate stress responses, four RNA-seq determinations were made in triplicate to allow pairwise comparisons of nitrogen stress under similar phosphate conditions and of phosphate stress under similar nitrogen conditions; these experiments are labeled as described in Figure 1 and Supplementary Figure 1 with, for example, NSRP designating the Nitrogen Stress Response under high phosphate and NSRp designating the Nitrogen Stress Response under low phosphate. Stress responses were defined as the sets of genes where expression changed two-fold or more between the stress condition and the non-stress condition. As indicated in Supplementary Figure 1B, different numbers of potential stress responsive genes might respond to the “same” stress under different experimental conditions.

**TABLE 1:** Distribution of Nitrogen and Phosphate responsive genes on the *S. meliloti* replicons.

	# annotated genes including ncRNAs	% total	NSR total	% of NSR total	NSR up	% of NSR up	NSR down	% of NSR down	PSR total	% of PSR total	PSR up	% of PSR up	PSR down	% of PSR down
Chromosome	4446	54	368	62	235	72	147	50	1263	62	444	49	828	73
pSymB	1977	24	139	23	61	19	81	28	455	22	297	33	159	14
pSymA	1830	22	90	15	30	9	63	22	318	16	170	19	149	13
Totals	8253		597		326		291		2036		911		1136	

*For each replicon, the number of annotated genes including non-coding RNAs is shown along with the percentage of the total # of genes that they represent. The total number of genes responding to nitrogen stress are indicated as well as the percentage of this group on each replicon. The number and percentages of nitrogen stress induced (NSR up) genes and nitrogen stress repressed (NSR down) genes are shown. Similar data for the phosphate stress responsive genes are also shown. When a gene was induced under one condition and repressed under another, it was counted twice; however, genes are only counted once for the NSR or PSR totals.*

**TABLE 2:** Nitrogen and Phosphate gene groupings.

Cluster	Cluster Definition	Genes in cluster
N.I	Responds to N stress during both P stress and non-stress conditions. NSRP AND NSRp	30
N.II	Responds to N stress only during P non-stress conditions. NSRP AND NOT NSRp	252
N.III	Responds to N stress only during P stress conditions. NOT NSRP AND NSRp	315
P.I	Responds to P stress during both N stress and non-stress conditions. PSRN AND PSRn	703
P.II	Responds to P stress only during N non-stress conditions. PSRN AND NOT PSRn	895
P.III	Responds to P stress only during N stress conditions. NOT PSRN AND PSRn	438

*Each cluster is defined and the number of genes assigned to each cluster is shown.*

Specifically, we measured RNA levels using RNA-seq under four conditions, minimal MOPS medium containing ammonium/2mM phosphate [NP], ammonium/50 μM phosphate [Np], glutamate/2mM phosphate [nP], and glutamate/50 μM phosphate [np]. Pairwise comparisons were made (Supplementary Table 1) to identify genes with significant (*q* ≤ 0.01) two-fold changes in abundance of RNA in response to nitrogen or phosphate availability. Genes were grouped based on their change in response to the primary nutrient, either nitrogen or phosphate, and then sorted further based on the availability of the second nutrient. NSRP or PSRN groups contained genes where there were significant changes in expression in response to limitation of one nutrient when the other nutrient was sufficient. NSRp or PSRn groups contained genes that changed in response to a nutrient limitation when the other nutrient was also limited. “Core NSR” or “Core PSR” groups contained genes where expression changed in response to one nutrient independent of the status of the other nutrient, e.g., induced >2× by nitrogen stress under *both* phosphate conditions. The results of this analysis are shown graphically in Figure 1A for the nitrogen stress responses NSRP and NSRp and in Figure 1B for the phosphate stress responses PSRN and PSRn. Genes that were upregulated when both nitrogen and phosphate were limited could include genes that are integral to the NSR or the PSR and specifically necessary to cope with either stress, but they could also include genes that are important for coping with a generalized reaction, such as decreased growth rate or a response to generalized nutrient stress.

## Results and Discussion

### Overview

Overall, 597 genes had significant expression changes in response to nitrogen limitation (Supplementary Table 2) and 2036 genes had significant expression changes in response to phosphate limitation (Supplementary Table 3). When comparing the nitrogen and phosphate groupings, 493 genes were in common and had significantly changed expression in response to limitation of each nutrient (Supplementary Table 4). With respect to the direction of gene regulation, 53% of nitrogen responsive genes were upregulated in response to nitrogen deficient conditions and 47% were downregulated, whereas only 46% of phosphate responsive genes were upregulated in response to phosphate deficiency and 54% were downregulated. A heat map of the replicate experiments that led to these conclusions illustrates the variation in expression observed (Supplementary Figure 2).

In a microarray transcriptome study by Yurgel et al. (2013), 132 genes in wild type Rm1021 were identified as responding to nitrogen using the criterion of a two-fold change at *p* ≤ 0.05. Of those, 49 were identified in this study. A microarray study by Krol and Becker (2004) identified 234 genes that were either phosphate- or PhoB-dependent using the criterion of a three-fold change at *p* ≤ 0.05. 167 of these were also identified in this study. The appearance of previously identified genes in this study indicated that the bacteria responded to the nutrient stress in a consistent manner, while the inability to identify all the genes from previous studies most likely was due to experimental differences and a more stringent statistical limit. An important difference could be in the strain genetics. This study used Rm1021 *pstC*+ whereas Yurgel et al. used Rm1021 for their nitrogen stress transcriptome. Rm1021 contains a truncated *pstC* and cannot use the PstSCAB, a high-affinity, high-velocity phosphate transport system found in related strains (Yuan et al., 2006a). Krol and Becker’s phosphate stress microarray involved Rm2011, a closely related *pstC*+ strain (Wais et al., 2002). Other differences between the studies included the media used, culture density at time of collection, and factors such as fold change cutoff and Q value. The novelty in this transcriptome compared to the previous studies is that not only were nitrogen or phosphate responsive genes identified, but their sensitivity to the other nutrient was also characterized, enabling analysis of genome-wide changes as a function of two variables and identification of genes or processes that were coordinately regulated by both stresses.

In total, 597 genes responded to nitrogen limitation under some condition (Figure 1A). The typically measured “NSR,” NSRP, consisting of genes whose expression responded to nitrogen limitation when cells had sufficient phosphate, contained 282 genes or 47% of the total NSR genes. The NSRp group, which responded to limited nitrogen when phosphate was also limited, contained 345 or 58% of total nitrogen responsive genes. 30 genes (5%) had significant expression changes under both phosphate sufficient and phosphate deficient conditions and made up the “Core NSR” group described above. Of the 2036 genes that responded to phosphate limitation, 1598 genes or 78% responded to phosphate when nitrogen was sufficient and were placed in the PSRN group. The remaining 1141 genes or 56% were assigned to the PSRn because they responded to phosphate limitation when nitrogen was also limiting (Figure 1B). 703 genes or 34% were phosphate responsive during both nitrogen conditions and were included in the “Core PSR.” Most notable in both analyses was the number of genes that responded to stress under one condition but not the other. For phosphate stress, the percentage of the 2036 genes that responded under high nitrogen but not low nitrogen or vice versa was 1336, or 66% of the total. For nitrogen stress, 567 of the 597 genes or 95%, responded under one but not both conditions. Defining a gene as nitrogen stress responsive seems therefore to have little predictive power when applied to these different contexts.

In these experiments, phosphate deficiency influenced more than three times as many genes as nitrogen deficiency. The data indicate that expression of a substantial fraction of genes were influenced by both nutrients as the primary stress condition, identifiable by the presence of these genes in both the nitrogen-responsive and phosphate-responsive tables (Supplementary Table 4). This implies either that many genes can be part of both an NSR and a PSR, or that stress itself influenced their expression as part of a global response. We observed that phosphate limitation led to many changes in translational proteins, a systemic response that was less prominent during the nitrogen limitation.

### Gene Expression Patterns Differ on the Three *S. meliloti* Replicons

**FIGURE 2:**
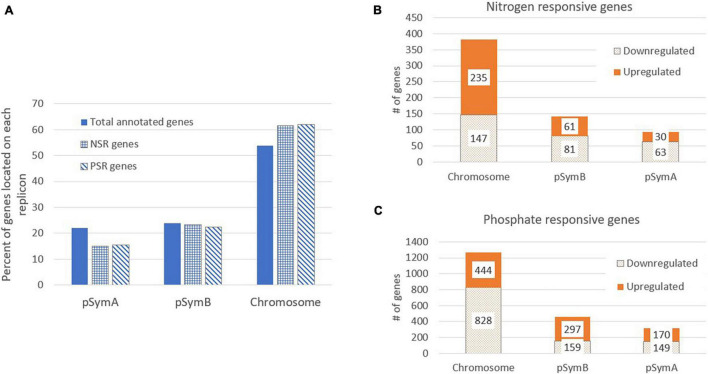
Distribution of Nitrogen and Phosphate responsive genes on the *S. meliloti* replicons. **(A)** The percent of *S. meliloti* genes located on each replicon is shown for total annotated genes (solid bars), the nitrogen responsive genes (NSR) (checkered bars) and the phosphate responsive genes (PSR) (striped bars). The number of genes with significant expression changes in response to nitrogen stress **(B)** or phosphate stress **(C)** are indicated for each replicon. Each bar is split and shows the number of upregulated (solid bars) and downregulated (dotted bars) genes per replicon.

*Sinorhizobium meliloti* Rm1021 contains three large replicons: a chromosome (3.7 Mb) and two megaplasmids, pSymA (1.4 Mb) and pSymB (1.7 Mb) (Galibert et al., 2001; Table 1). Table 1 and Figure 2 show an analysis of the number of genes on each replicon that are induced or repressed by nitrogen stress (Figure 2B) and phosphate stress (Figure 2C). The three replicons contain 54, 22, and 24% of the total annotated genes, but the genes responding to the stresses and the way that they respond are not proportionately distributed. In particular, the genes that respond to nitrogen stress are more common on the chromosome and less common on the pSymA megaplasmid. This is most pronounced for the genes induced by nitrogen stress, as can be seen in Table 1 where the proportion of N-inducible genes on pSymA is less than half of what might be expected based on random distribution throughout the replicons. A similar trend is not seen for pSymA genes repressed by nitrogen stress. In contrast, the fraction of genes on both plasmids that are repressed during phosphate stress is lower than expected.

### Nitrogen Responsive Genes

Supplementary Table 2 contains the list of 597 significantly changed nitrogen responsive genes. The genes with the highest induction ratios in response to nitrogen deficiency had all been identified previously (Yurgel et al., 2013), but measuring the phosphate modulation of these nitrogen responsive genes was novel and provided insight into how the stress response pathways were sensitive to the status of the other nutrient. The discussion of specific genes below is structured around the Venn diagrams in Figure 1 and the clusters defined in Table 2. For both the NSR and PSR, we define a set of Core genes (N.I and P.I), and two sets of genes that were included in the stress response under one condition but not the other, i.e., N.II corresponds to the NSRP, while N.III corresponds to the NSRp. It is important to realize that, as strictly defined as the Venn diagrams make these distinctions appear, these boundaries are numerical and not conceptual. Within each cluster we have tried to focus on genes that seemed most interesting in terms of their previously described regulation by nitrogen or phosphate limitation, and to illustrate how the availability of one nutrient might affect expression of genes also controlled by the other. Supplementary Table 5 shows the number of genes in major classification categories (as seen in Supplementary Tables 2, 3 in the “classification” column) for the NSR and PSR and the number of genes in each category that are up- or downregulated. For the set of genes described as N.III, which corresponds to the NSRp, it is important to recognize that these genes, which comprise the largest of the three sets, had not generally been defined as NSR genes in previous analyses that did not include the additional dimension of phosphate limitation. Our analysis thus evaluated stress response pathways individually, but also links the responses, demonstrating the large multi-dimensional aspect of gene regulation when more than one stress was applied.

### Core Nitrogen Stress Response Genes

We defined the Core NSR group N.I as 30 genes that had significant expression changes in response to nitrogen limitation under both phosphate sufficient and phosphate limiting conditions. Given the limits of the experimental design, we consider these to “always” be part of the NSR (Figure 1, Table 2, and Supplementary Table 2). *glnII* and *dctA* were both highly induced. *glnII* is well known as a nitrogen-regulated gene although occurrence of induction during conditions of either phosphate sufficiency or deficiency had not been reported. The induction of *glnII* may indicate that it is very important during nitrogen limitation and needs to be expressed regardless of the phosphate status, although phosphate still had a substantial effect on the level of induction— *glnII* was induced 28-fold in high phosphate and only 2.9-fold in limiting phosphate. In a pattern that probably contributes further to the modulation of the GSII protein in response to low nitrogen, *gstI*, an inhibitor of glutamine synthetase II translation (Napolitani et al., 2004), was repressed four-fold by low nitrogen, but only when phosphate was sufficient. It was somewhat surprising to find *dctA* in this cluster. DctA is a dicarboxylic acid transporter that is needed for growth on C4 dicarboxylates like malate, succinate, fumarate and aspartate (Yurgel and Kahn, 2004, 2005). DctA is essential for nitrogen fixation during symbiosis, probably to acquire the carbon compounds needed for generating energy and reductant (Yurgel and Kahn, 2004). Like *glnII*, *dctA* expression was much higher under phosphate sufficiency (12.8×) than in limiting phosphate (2.6×).

### Nitrogen Stress Response Genes During Phosphate Sufficiency

The NSR group N.II contained 252 genes that responded to nitrogen limitation only when phosphate was sufficient (Figure 1, Table 2, and Supplementary Table 2). Under high phosphate, 88 of these genes were upregulated by nitrogen stress and 164 were downregulated. Interestingly, genes like *glnK* and *amtB*, which were highly induced by nitrogen limitation during phosphate sufficiency (>100×), were induced less than 2× when cells were grown on glutamate during phosphate limitation. *amtB* codes for an ammonium uptake transporter that is often found in an operon with *glnK* and both GlnK and GlnB P_*II*_ proteins can activate *amtB* expression. *glnK* and *amtB* had previously been shown to be induced during nitrogen stress (Yurgel et al., 2013). Identification of these genes as highly induced by nitrogen stress in high phosphate is consistent with other observations in the literature but the impact of phosphate stress on their expression had not been described. Other highly induced genes in this category included *nrtA*, encoding a nitrate reductase, and *rhtA*, encoding a protein involved in rhizobactin regulation. Rhizobactin is a siderophore that can bind iron and other metals used as cofactors for various proteins, suggesting that metal metabolism and acquisition may be affected by nitrogen availability.

Changes in expression of N.II genes occurred only during phosphate sufficiency suggesting that the additional phosphate stress rendered these changes less important to the cell even though nitrogen was still limited. Many of these genes have transport-related functions. For example, the genes in *hmuPSTUV*, an operon that codes for a hemin import system, were upregulated, as was *smhR*, an outer membrane hemin binding protein. In contrast, the unlinked *hemA, hemB, hemE, hemF, hemH* and *ctaA* genes, which code for proteins involved in heme biosynthesis, were downregulated. The upregulation of hemin transport and downregulation of heme biosynthesis suggests that under these conditions, the cell prefers to import heme rather than to generate it *de novo*. This example illustrates how a cell can conserve energy and resources by repressing biosynthesis of certain molecules, but still tries to obtain the molecules from its environment, potentially satisfying both the greed and fear motivators. The *livH-livK* operon, which codes for a branched chain amino acid transporter, was highly induced by nitrogen stress. The corresponding *bra* operon is important in the symbiosis between *Rhizobium leguminosarum* and the pea plant and when inactivated results in symbiotic auxotrophy. In the symbiosis between *S. meliloti* and alfalfa, the symbiotic auxotrophy phenotype is not observed when these transporters are inactivated (Prell et al., 2010). Induction of these transporters in this context suggests that these systems are beneficial during nitrogen stress, but phosphate is necessary to use them effectively, potentially because resources are needed elsewhere or to be consistent with other changes imposed by the additional phosphate stress. The *SMc02344-SMc02346* genes, which code for components of a glycine-betaine and choline ABC transporter, were also among the most highly induced genes. This could indicate that these compounds were a preferred source of nitrogen for the cell during nitrogen limitation, or that they were induced to take advantage of the possibility that these osmolytes could play a larger role in adaptation during nitrogen stress.

### Nitrogen Stress Response Genes During Phosphate Deficiency

The N.III group contained 315 genes that showed significant expression changes in response to nitrogen limitation only when phosphate was also limiting (Figure 1, Table 2, and Supplementary Table 2). Of these, 209 were upregulated by low nitrogen and 106 were downregulated. Most genes in this group had not been identified as part of an NSR. Showing that there is a nitrogen stress response under low phosphate conditions that is distinct from the classical NSR is significant because not only does it include additional genes in the bacterial NSR, but it shows that defining an NSR is very context dependent.

One of the upregulated genes in the N.III group is a putative alkaline phosphatase, *SMc03243*. The presence of a putative alkaline phosphatase that responded to nitrogen limitation was interesting but not entirely unexpected because previous studies had shown that P_*II*_ proteins can influence expression of genes in the phosphate regulon (Hagberg et al., 2016). While our earlier work had not identified *SMc03243* as significantly responsive to nitrogen (Yurgel et al., 2013), the added P stress and strain differences, specifically the introduction of the *pstC*+ allele into the Rm1021 *pstC*+ strain used here, may have allowed *SMc03243* to respond differently than it does in Rm1021, which has the truncated *pstC* gene.

### Phosphate Responsive Genes

In total, 2036 genes, or about one third of all *S. meliloti* genes, had significant expression changes when comparing low and high phosphate conditions (Supplementary Table 3). This was nearly four times the number of genes that responded to limited nitrogen. The 167 phosphate-responsive genes that were also found in the microarray experiments of Krol and Becker (2004) had various patterns of expression with respect to the influence of nitrogen. Choudhary et al. (2020) defined the regulon of PhoR/PhoB in *E. coli* which includes 170 genes. Homologous genes in *S. meliloti* responded to phosphate which was expected due to the conserved PhoRB two-component system involved in regulating the PSR in both bacteria. These genes also had various patterns of expression with respect to nitrogen influence, but the majority were in the Core PSR or PSRN groups.

### Core Phosphate Stress Response Genes

The Core PSR group P.I contained 703 genes whose expression significantly changed in response to phosphate limitation under both nitrogen conditions (Figure 1, Table 2, and Supplementary Table 3). This large number contrasts with the 30 genes in the Core N.I NSR group. The difference suggests that phosphate limitation may be a greater strain on the cell than nitrogen limitation, and that a larger set of genes must be either induced or repressed to maintain critical cell functions or to restructure processes to use the available phosphate most efficiently. Of the 703 genes, 351 were upregulated and 343 were downregulated. To our surprise, nine mRNAs were significantly induced in one nitrogen condition and repressed in the other – a result that met the criteria for classification to the Core PSR but was unusual when considered from a viewpoint in which the PSR describes sets of “phosphate induced” or “phosphate repressed” genes that act to counter the cellular effect of phosphate stress.

Of the 100 genes most highly induced or repressed in response to phosphate stress, about 70% belong to the Core PSR. Many of these had been previously characterized as core components of the PSR. The *pho, pst*, and *phn* operons are involved in phosphate transport or phosphonate uptake and metabolism and are known to be induced by phosphate stress (Voegele et al., 1997; Parker et al., 1999; Yuan et al., 2006a; Choudhary et al., 2020). Induction of these operons allows cells to catabolize phosphorous-containing molecules in order to transfer phosphate into molecules with a higher priority.

*phoB, phoX*, and *phoU* were among the most highly induced. The PhoB response regulator controls activation of the PSR in association with its sensor kinase, PhoR. Induction of *phoB* should permit a greater dynamic range in transducing the phosphate deficiency signal into responsive gene and protein expression changes. PhoX is an alkaline phosphatase whose gene was induced 254-fold by phosphate stress in these experiments. PhoX is one of several extracellular phosphatases that degrade phosphate esters to liberate phosphate. *SMc03243*, which codes for a putative alkaline phosphatase, was among the most induced genes. Its strong induction during phosphate limitation is consistent with its annotation as a putative alkaline phosphatase and indicates that the cell uses this phosphatase in addition to PhoX. Also in this group are genes that could be used in releasing phosphate from phosphonate and phytate. PhoU is thought to be a PSR regulatory protein but its precise role is unclear (Lubin et al., 2016). Among the most repressed genes was *pit1* which encodes a low affinity phosphate transporter, presumably reflecting its lack of utility under these conditions.

Other genes that were highly induced in the Core PSR P.I group were *SMc04316, SMc04317, wgeA, btaA* and *btaB. SMc04316* and *SMc04317* are in an operon that has a Pho box in its promoter region and are predicted to encode iron transport proteins (Yuan et al., 2006b). *wgeA* codes for a galactoglucan (EPSII) biosynthetic protein that is regulated in part by PhoB and induced under low phosphate conditions (McIntosh et al., 2008). *btaA* and *btaB* encode proteins that produce diacylglycerol-*N,N,N*-trimethylhomoserine (DGTS), a phosphorous-free lipid that can be incorporated into the membrane during phosphate stress (López-Lara et al., 2005). DGTS and related lipids are a major fraction of lipids induced by phosphate starvation (Geiger et al., 1999). Inducing *wgeA, btaA* and *btaB* may help restructure the cell envelope by replacing phosphate-containing molecules, like phospholipids, with phosphate-free molecules or to protect the cell during stress via increased polysaccharide production.

Of the 36 messages in the Core PSR coding for ribosomal proteins (*rps, rpm, and rpl*), all were repressed, with 30 downregulated more than 4×. Ten tRNA synthetases were repressed between 2.5- and 5-fold. This suggests phosphate stress significantly downregulates protein synthesis by interfering with protein translation.

### Phosphate Stress Response Genes During Nitrogen Sufficiency

The P.II group contained 895 genes that had significant expression changes in response to phosphate stress only when nitrogen was sufficient (Figure 1, Table 2, and Supplementary Table 3). In particular, RNAs corresponding to the protein synthesis apparatus, including six Rpm proteins, seven Rps proteins, four Rpl proteins, and several other proteins annotated as important in ribosome function are in this group. Three tRNA molecules, gly_TCC, thr_CGT, and asp_GTC are repressed 9.8, 9.5, and 5.1× respectively. The glycine codon is not rare in *S. meliloti* and the threonine and aspartate codons are used extensively^1^. The P.II group also includes seven downregulated tRNA synthetases (including *thrS* and *glyQ*) and six tRNA modification activities, including *gatB*, which amidates aspartate and glutamate on charged tRNAs to yield asparagine and glutamine tRNAs. Thus, phosphate stress in the presence of ammonium has the potential to downregulate protein synthesis through direct interference with the translational machinery.

Amino acid biosynthetic gene expression also changed. *ilvA, ilvD1, ilvD2, and ilvE1, leuA1, leuB, leuD, and leuS* and *hisBHF, hisC2, hisD1 and hisZS* are independently regulated genes or parts of operons that are involved in branched chain amino acid, leucine, and histidine synthesis, respectively. These were all significantly downregulated in response to phosphate limitation only when nitrogen was sufficient. The downregulation of these amino acid-related genes by phosphate limitation was consistent with previous transcriptome data in *S. meliloti* (Krol and Becker, 2004) and with phosphorous limitation experiments in *E. coli* (Van Bogelen et al., 1996) and might reflect reduced demand for these amino acids in phosphate limited cells. Downregulation of these genes may potentially be metabolically complemented by induction of transporters such as *Smc02832* which is categorized as a putative taurine, valine, isoleucine and leucine ABC transporter and also in the P.II group.

The *nuoA1B1C1D1E1, nuoG1I, nuoK1LN* genes, which code for various subunits of an NADH-quinone oxidoreductase and are clustered in a regulon on the chromosome, and the *nuoI2* gene, which is in a separate cluster on pSymA, were downregulated during phosphate stress when nitrogen was sufficient. *ctaCDBGE*, an operon that codes for synthesis and assembly of a cytochrome c oxidase, was also downregulated. These changes are consistent with a change in the energy and carbon metabolism of the cell during phosphate stress.

### Phosphate Stress Response Genes During Nitrogen Deficiency

The P.III group contained 438 genes whose expression changed at least two-fold in low phosphate, but only when nitrogen was also limited (Figure 1, Table 2, and Supplementary Table 3). 320 of these genes were upregulated and 118 were downregulated by phosphate stress. These genes are “new” phosphate stress response genes, in the sense that they add to the traditional PSR which only considered genes whose expression changed under the standard condition of limiting phosphate in an otherwise complete medium. One interpretation of the appearance of this new set is that when nitrogen is sufficient their baseline expression does not need to be modified substantially to adapt.

*phoR, phoH*, and *phnN* were among the most highly induced genes in response to phosphate stress, but these were induced only during nitrogen limitation. It is expected that these genes would be induced based on their role in phosphate transport and regulation. A few other genes also responding to phosphate stress during nitrogen deficiency were *katB* and the cold shock family RNA binding protein genes *cspA1, cspA3*, and *cspA7.* KatB encodes one of *S. meliloti*’s catalase/peroxidase enzymes and helps detoxify oxidative stress molecules. The cold shock family of stress proteins have protective roles during various stress events, such as low temperature and salt stress, and they also have roles in symbiotic development (O’Connell and Thomashow, 2000; Price, 2017). Increased expression of these genes indicates that cell protection and detoxification are important processes during nutrient stress and suggests the cell is reducing non-essential processes that may leave the cells vulnerable to other stresses imposed by predators, competing microorganisms, or toxic molecules in addition to scavenging for nutrients.

### Genes Responding to Both Nitrogen Stress and Phosphate Stress

In total, 493 genes are present in both the nitrogen-responsive gene table and the phosphate-responsive gene table (Supplementary Table 4) and will be referred to as “dual-responsive genes.” In some cases, a gene was upregulated or downregulated in response to both nutrient limitations, but 289 of these genes, or 58%, were upregulated in response to limiting one nutrient and downregulated in response to limiting the other nutrient. Of these, nearly 2/3 of the genes were upregulated during nitrogen limitation and downregulated by phosphate limitation, demonstrating that the changes in GS mRNA abundance as a function of both nitrogen and phosphate availability, as previously discussed in the introduction and documented in Hagberg et al. (2016) are not unique.

One gene that responded significantly to both nitrogen and phosphate stress was *SMc03243*, the putative alkaline phosphatase discussed previously. *SMc03243* was highly induced by phosphate limitation under both nitrogen conditions, but the induction was two-fold higher when nitrogen was limited compared to when it was sufficient. The *SMc03243* expression data and analysis illustrates how genes can be “turned on” to various degrees based on the number and type of stresses, further indicating an integrated response.

*glnII* expression was also dual responsive. Upregulation of *glnII* during nitrogen stress was expected (Yurgel et al., 2010). By using reporter fusions and immunoblot assays, we showed earlier that *glnII* induction by nitrogen limitation was lower when phosphate was limited (Hagberg et al., 2016). The data here showed that *glnII* mRNA was induced during nitrogen stress in both phosphate conditions, but the induction ratio was seven times greater when phosphate was sufficient compared to deficient. *gstI*, which codes for a GSII translational inhibitor located adjacent to *glnII* and transcribed divergently (Spinosa et al., 2000), was regulated in a way that counters expression of *glnII*, i.e., it was repressed by low nitrogen. The reciprocal regulation of these two genes is thought to enhance the range of GSII regulation (Spinosa et al., 2000; Napolitani et al., 2004). The response of *glnII* and *gstI* to phosphate stress was also inverted—during nitrogen stress, *glnII* expression was reduced by concurrent phosphate stress whereas *gstI* expression was enhanced by phosphate stress. The coordinated and reciprocal expression patterns of *glnII* and *gstI* indicate that phosphate stress decreased induction of GSII, a central component of the NSR, presumably to direct resources to both stress response pathways. Another interpretation is that phosphate stress has a more detrimental impact on the cell, and the standard NSR response pathway is downregulated to allow the cell to prioritize its response to phosphate stress.

Expression of a heme transport system encoded by an operon containing *hmuS* and *hmuT*, was upregulated by nitrogen stress, but downregulated when phosphate stress was added. *SMc01515* and *SMc01517*, uncharacterized genes situated just upstream of the *hmu* operon, were also upregulated by nitrogen stress and downregulated with simultaneous phosphate stress, suggesting that this region was regulated as a unit. In response to nitrogen limitation, *hmuS* was induced 2.2-fold when phosphate was sufficient but was repressed 3.6-fold when phosphate was limited. This pattern shows that phosphate stress can not only suppress the level of induction or repression that is caused by nitrogen stress but can even reverse the direction of the change from induction to repression or vice versa. 36 other dual-responsive genes had a reverse in direction of the induced change. Many of the genes that followed this pattern encoded transporters, suggesting that when nitrogen is hard to find, importing metabolites has a relatively higher priority than biosynthesis. However, the general downregulation of these transporters during phosphate stress suggests that this strategy is not as appropriate when phosphate is scarce. This could be because the two stresses are usually found in different contexts; nitrogen stress in a situation where other nutrients may still be available but phosphate stress when there is a general lack of external resources. Another possibility is that the demand for molecules decreases when the cell is scavenging for phosphate to maintain critical cell functions, and this impacts processes like import.

Members of the *liv* operon, which encode a branched chain (leucine, isoleucine, and valine) ABC transporter, were upregulated by nitrogen stress and downregulated by phosphate stress. The *liv* genes are known to be induced by nitrogen limitation, and Yurgel et al. (2013) suggested that they were regulated by GlnB/GlnK and another, unknown regulatory component. Sobrero et al. (2012) demonstrated that the Liv system and other amino acid transporters are negatively controlled by the RNA chaperone Hfq. Expression of *SMc02344-Smc02346*, encoding a putative glycine-betaine and choline transporter, also involves Hfq-dependent riboregulation (Barra-Bily et al., 2010; Sobrero et al., 2012). Like the branched chain amino acid transporters, this operon was upregulated during nitrogen stress and downregulated during phosphate stress. The identification of these nitrogen and phosphate responsive operons as being influenced by Hfq suggests that Hfq may be a major regulatory component that is involved in integrating the nitrogen and phosphate stress signals, perhaps by mediating sRNA-mRNA interactions.

### sRNAs and Hfq

**FIGURE 3:**
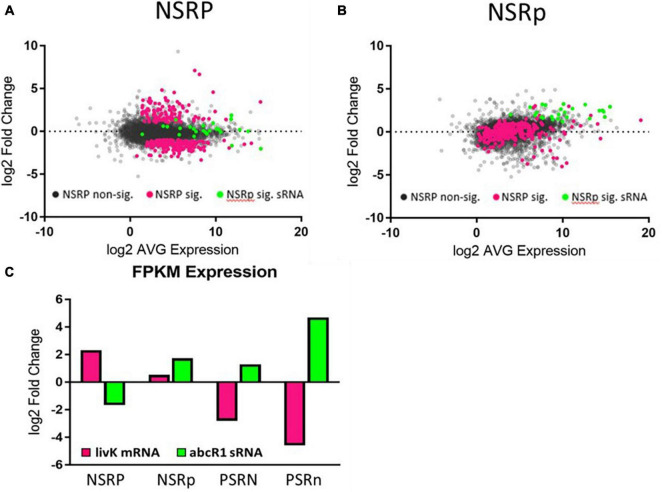
Nitrogen stress affects sRNA expression differently under high and low phosphate. Graphs showing the changes in expression of genes in the **(A)** NSRP and **(B)** NSRp. Gray dots represent non-significant gene expression changes. Pink dots represent the set of genes that were significantly changed in the NSRP. Green dots represent the sRNAs that were significantly changed in the NSRp. The expression patterns of each group can be seen changing between the high P condition **(A)** and the low P condition **(B)**. **(C)** The levels of *livK* and *abcR1* expression under the various stress conditions demonstrate the different expression patterns of an mRNA and its regulatory sRNA.

Hfq is an RNA chaperone that interacts with sRNAs used to regulate translation of expressed genes. Hfq often affects sRNA binding to the 5′ UTR of mRNAs, thereby affecting 30S ribosomal subunit binding to this region during the initiation of translation (Vytvytska et al., 2000; Soper et al., 2010). The abundance of 45 sRNAs was significantly altered in our experiment, and 14 of these are known to interact with Hfq (Torres-Quesada et al., 2014). These sRNAs had some of the largest fold changes between the stressed and non-stressed conditions, with levels of induction in the range of the most highly induced protein-encoding genes associated with nitrogen or phosphate stress. This suggests that these sRNAs play a major role in regulating translation of transcripts sensitive to nutrient stress. The abundance of a majority (19) of sRNAs responding to nitrogen were significantly changed only in the phosphate deficient condition (N.III) (Figure 3). Only 5 sRNAs responded to nitrogen during phosphate sufficiency (N.II). An additional 4 sRNAs responded to nitrogen during both phosphate sufficiency and deficiency (N.I). Phosphate stress altered the expression of 13 sRNAs during both nitrogen conditions (P.I). Only 8 sRNAs responded to phosphate limitation during nitrogen sufficiency (P.II) while 16 sRNAs were phosphate responsive during nitrogen deficiency (P.III). These data indicate phosphate stress influences sRNA abundance more than nitrogen stress does, consistent with the data observed for mRNA abundance changes.

One highly induced sRNA was made from *abcR1* and was induced during nitrogen limitation when phosphate was also limited. *abcR1* is known to negatively regulate expression of the *livK* gene with the aid of Hfq (Torres-Quesada et al., 2014). *livK* expression was induced by nitrogen limitation, but then strongly repressed by the addition of phosphate stress (Figure 3C). The induction of the *abcR1* sRNA during the double stressed condition could act to inhibit translation of the *livK* transcripts, which were also being repressed, suggesting that while LivK is useful during nitrogen stress, it might not be needed or was inefficient during phosphate stress, and that repression of LivK transcription and translation by low phosphate allows resources to be directed elsewhere.

In addition to the *livK* example linking a nutrient stress-induced sRNA with Hfq, regulation of the *E. coli rpoS* gene by both PhoB and Hfq has been observed (Ruiz and Silhavy, 2003). In this case, *rpoS* translation is increased during phosphate limitation by a PhoB-induced sRNA. Hfq mediates the sRNA-*rpoS* mRNA interaction, enhancing translation of the *rpoS* message, and demonstrates how PhoB and Hfq can both be involved in regulating a gene. There is no *rpoS* in *S. meliloti*, but Hfq has been linked to numerous changes involved in the metabolism of nitrogen-containing compounds, transporter systems, general stress, mobility, and membrane components (Gao et al., 2010; Torres-Quesada et al., 2014). One third of the dual-responsive genes in this study were previously identified as having significant expression changes in an *S. meliloti hfq* mutant compared to wild type (Barra-Bily et al., 2010; Gao et al., 2010; Torres-Quesada et al., 2010, 2014; Sobrero et al., 2012). The possibility that Hfq-mediated sRNAs have a role in these processes correlates with the large number of transport genes identified as being dual-responsive to nitrogen and phosphate stresses. Hfq may be interacting with sRNAs to alter translation of a class of target transcripts and help integrate and fine-tune the response to these stresses.

### Mapped Networks

**FIGURE 4:**
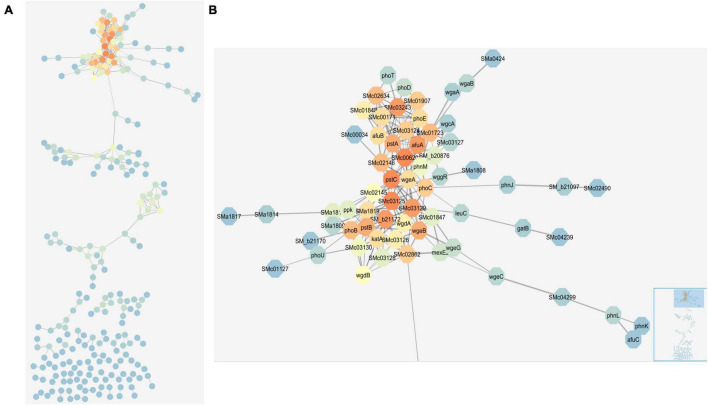
Network map of connected genes based on weighted co-expression analysis. Network map created in Cytoscape depicting genes that are linked based on their expression patterns and correlation with each other. **(A)** Shows the full network map. **(B)** Shows the large group at the top of the map including many core phosphate stress response genes. Colors represent degrees and depict how connected a gene is to other genes in the map. The color scheme starts with blue as the least connected (fewest degrees) and flows through green to yellow to orange as the most connected (highest degrees). The bottom right inset in panel **(B)** highlights the area of the full network map that is enlarged.

Weighted co-expression analysis using R bioinformatics software and visualization with Cytoscape (Shannon et al., 2003) allowed us to construct a map of co-expressing genes (Figure 4 and Supplementary Figure 3). The co-expression analysis was able to connect genes that had similar patterns of expression change across the four media conditions. For example, core components of the PSR, such as *phoB* and the *pst* operon, respond similarly to the four conditions and were connected in the network. The largest group in the network included *phoB* and many of the highly induced phosphate responsive genes described above, but also genes that met the covariance criteria which did not stand out in the pairwise analysis, such as *SMc00620, SMc01723*, and *SMc02862* (Figure 4B). The second largest network featured chemotaxis and mobility genes, which also form a well-documented regulon that responded coordinately to these nutrient stresses. In *E. coli*, the PhoB regulon also contains the chemotaxis genes along with the expected phosphate transport systems (Choudhary et al., 2020).

The visualized connections between the large group containing *phoB, phoCDET, pstSCAB, phoU*, and other genes involved in phosphate stress represent some of the most upregulated genes during phosphate stress with no significant up or down regulation during nitrogen stress. The highly connected nature of these genes was expected—they have been consistently identified and characterized as being part of the PSR (Al-Niemi et al., 1997; Krol and Becker, 2004). Also connected in this group were some regulators of exopolysaccharide synthesis. Exopolysaccharide synthesis is changed based on phosphate availability, so the connection of these genes with other phosphate genes further validates the network map.

Surprisingly, given the central importance assigned to the *phoBR* two-component regulatory system in phosphate stress regulation, these genes were not the “most connected” in the network as indicated by the degrees parameter. This distinction went to *SMc00620*, with 20 connections, many more than the 12 for *phoB*. *SMc00620* is a hypothetical protein which a BLAST search indicates is likely to be a phytase type of phosphodiesterase. Because of its large number of direct connections and co-expressing genes, *SMc00620* may be worth exploring further by mutation to determine whether it regulates or is regulated by this unusually large number of other PSR-associated genes. If this gene does produce a phytase, it is conceivable that it is upregulated during phosphate stress to release phosphate from plant-produced phytate compounds that may be in the rhizosphere. Phytases may not be common in enteric bacteria due to the lack of phytate in their natural environments and thus, not commonly observed in traditional PSRs of model bacteria like *E. coli.*

The network map brought attention to genes with many connections that may have been overlooked in the pairwise analysis due to their uncharacterized or putative nature and it provides candidates for further exploration within the stress response pathways. *SMc02862*, which is annotated as a *pit* transport system accessory protein, also has more links to other genes in this cluster than *phoB* (14 > 12). The *pit* gene, *SMc02861*, was downregulated by phosphate stress, but only *SMc02862* was included in the co-expression network. The Pit transport system is a low-affinity phosphate transport system whose regulation by phosphate stress is known to be reciprocal to that of the high-affinity Pst and Pho transporters (Voegele et al., 1997).

### The BK *pstC*+ Strain Has Far Fewer Significant Expression Changes Than 1021 *pstC*+

**FIGURE 5:**
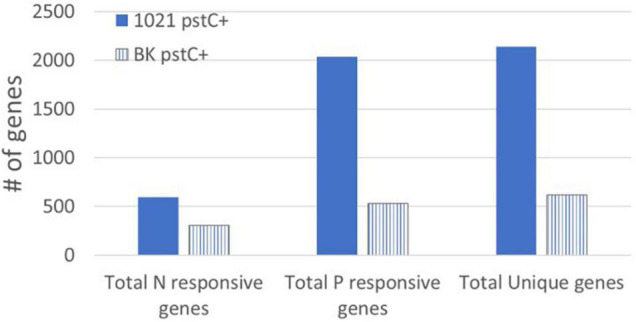
Genes responding to nitrogen and phosphate stress in the BK *pstC*^+^ strain. The total number of genes responding to nitrogen stress (left) and phosphate stress (center) in the 1021 *pstC*+ (solid bars) and BK *pstC*+ (striped bars) strains. The total number of unique genes (right) that changed in response to at least one nutrient stress is also shown for each strain.

The P_*II*_ proteins, GlnB and GlnK, were originally identified as important components of the NSR signal transduction pathway, influencing both transcriptional and post-translational responses to low nitrogen. We analyzed the *glnB^–^glnK^–^* strain, BK *pstC*+, in addition to the 1021 *pstC*+ strain for gene expression changes that resulted from nitrogen and/or phosphate stress. The most striking observation was that expression of only 620 genes was altered significantly by nitrogen stress, phosphate stress, or both (Figure 5 and Supplementary Table 6). This number, while substantial, was far less than the 2140 stress responsive genes identified in 1021 *pstC*+ where the P_*II*_ proteins are intact. The lack of expression changes in BK *pstC*+ compared to 1021 *pstC*+ demonstrates the vital regulatory role that the P_*II*_ proteins play within the cell and reinforces the idea that their role is not limited to the NSR. Two scenarios could explain the lower number of gene expression changes in BK *pstC*+. One is that the slow growth of the BK *pstC*+ strain (Yurgel et al., 2010) may allow it to cope with stress by using smaller changes in gene expression due to a reduction in immediate demand for resources. In this scenario, the lack of the P_*II*_ proteins causes a change in growth rate that reduces the apparent stress, but the change is not directly coupled to the regulatory role of the P_*II*_ proteins.

A second scenario is that the mutant does not react normally to stress because of the lack of the P_*II*_ proteins and the direct disruption of stress signal transduction. The BK strains grew more slowly and had altered NSR regulation, as indicated by higher GS activity and expression than in wild type Rm1021 *pstC*+ during nitrogen sufficiency. However, the induction of GS activity and expression in the BK *pstC*+ deletion may not be the result of NtrC-P activity, but rather, mediated through PhoB-P, since a mutation in *phoB* lowers GS expression in the BK background (Hagberg et al., 2016). A link between GlnB/GlnK and PhoB has been observed, but the mechanism is unclear. Based on these observations, the nitrogen responsive changes observed in the BK *pstC*+ strain may not be caused by the classical GlnD→GlnB/GlnK→NtrB/NtrC pathway but may instead result from aberrant PhoB-P regulation or the involvement of some other regulatory circuit. The large number of genes in 1021 *pstC*+ that are phosphate responsive suggests that many of these are linked to a general stress response triggered by low phosphate but that this connection may not be phosphate specific. In the BK *pstC*+ strain, the general stress response may not be activated if GlnB and GlnK are indeed involved in regulation of general stress, resulting in slow growth and small colonies as the cell is extremely inefficient in coping with stress. In this interpretation, many of the “phosphate responsive genes” would not be changing as a function of phosphate limitation since they are inactive due to the lack of the P_*II*_ proteins and their probable role as part of the cell’s general stress response.

## Conclusion

*Sinorhizobium meliloti* is a model for an important group of alpha-proteobacteria that reduce nitrogen during symbiosis with legumes and contribute a substantial amount of fixed nitrogen to terrestrial ecosystems. To be able to initiate the symbiosis by infecting the plant roots, rhizobia must be able to survive in soil. This requires that they deal successfully with various stresses in the soil environment—stresses that can change with time and can differ from site to site. Bacterial stress response is a term that describes coordinated changes in gene expression that help the organisms deal with a stress. Historically, stress responses have been studied individually, which is a useful starting point but probably not representative of more natural conditions.

This work was aimed at characterizing the interaction and coordination of responses to limited nitrogen and limited phosphate, individually and together. The results show that gene expression in response to one stress may change drastically if another stress is applied at the same time. The change may be quantitative, where the degree of response differs in a pairwise comparison, but the direction of the response can reverse, and our analysis included genes where induction by N limitation with adequate P shifted to repression by N limitation in the deficient P condition and other repression/induction combinations. Reversal implies that a change useful under one condition might be a problem under the other. Phosphate stress generated a larger number of gene expression changes than nitrogen stress. Moreover, the response to phosphate limitation appeared to be dominant—when coping with both stresses, a set of genes that were induced during nitrogen stress were subsequently repressed by an additional phosphate stress. We interpret this to indicate that the phosphate stress used here was a larger burden on *S. meliloti*, and that dealing with the phosphate limitation had priority over dealing with the nitrogen limitation.

While the analysis of the data presented here depends to some extent on certain parameters used in the analysis, like the level of induction or repression used to group genes, we have carried out similar analyses using other parameters and the general conclusions are robust. Most notable was a striking and unexpected difference between the genes involved in the high phosphate NSRP and the low phosphate NSRp. 597 genes had two-fold induction or repression under at least one of these conditions representing about 10% of the genome. 252 genes were in the high phosphate NSRP and 315 in the low phosphate NSRp with only 30 genes common to both NSRs. Thus, while an NSR can be defined under each phosphate condition, the two NSRs are very different. It follows that designating a gene as nitrogen stress responsive is not as simple or useful as we thought it would be in designing this experiment. It also means that we are describing a major new class of NSR genes, those in NSRp, that were missed in previous analyses.

We do not know how generalizable the observations here may be. There are clearly some interactions between the NSRs and the PSRs and these involve regulation of genes that could be involved in other stress responses, like the iron stress response. Experiments designed to test the impact of limiting other nutrients would need to be done to assess these or to determine if the interactions are binary or related to some more general control pattern. Cross-regulation by regulatory proteins and the identification of imodulons or regulons composed of genes that are coordinately regulated, is a focus of recent research in other model organisms like *E. coli* (Sastry et al., 2019; Choudhary et al., 2020). Homologs of regulatory circuits in *S. meliloti* are often found in enteric bacteria, and our data are consistent with regulons found in *E. coli*. We would be surprised if the interactions between the NSR and the PSR are unique to *S. meliloti*.

The identification of sRNAs and mRNAs that are dual responsive to both nitrogen and phosphate stress contribute to an understanding of the coordination of stress responses. Our data show that regulation of individual genes in response to one stress can be altered drastically by imposing a second stress. Not only were novel genes identified as responding to each individual stress, but a group of genes were identified that only changed when both stresses were present. In addition, there was a subset of genes for which the direction of induction/repression was changed by the presence of a second stress, demonstrating the interconnected nature of the stress responses and how a single gene can have multiple layers of regulation that respond to the availability of more than one nutrient. Our data suggest that *S. meliloti* can prioritize stress pathways or specific processes based on nitrogen or phosphate availability.

An interesting group of genes was identified that responded significantly to both nitrogen and phosphate stress; the expression of many of these genes had been shown to be influenced by Hfq, an RNA binding protein and RNA chaperone. This association suggests that Hfq is involved in integrating the responses to various stress signals through its interaction with sRNAs and its role in translating mRNAs. Future experiments are needed to determine how the rhizobial proteome changes in response to similar growth conditions to determine if the mRNA responses to nitrogen and phosphate stress are reflected in protein abundance.

We demonstrated that the nitrogen and phosphate stress response pathways were not activated or inactivated independently or in only one dimension, but that they were instead connected with some groups of genes responding differently based on the status of a secondary stress. A key question this raises is how other nutrient stresses would affect the NSRs and PSRs. Cell envelope stress has been demonstrated to affect the PhoB regulon in *E. coli* (Choudhary et al., 2020). Changes in expression of heme biosynthesis and heme transport-related genes, and rhizobactin-related genes, suggest that iron stress regulation may be connected to nitrogen and phosphate stress regulation. While there might be interactions between nitrogen or phosphate stress and other stressors such as DNA damaging agents and oxidizing agents, there were only a few genes of this type, like *katA*, that were involved in the web of interactions we observed (Figure 4).

The multi-dimensional response to stresses is important in developing system-level knowledge of bacterial stress adaptation. This schematic of complex, inter-related stress responses might allow the development of bacteria able to function better under the relatively constrained conditions of industrial fermenter production or in naturally constrained environments. For the rhizobia, this could lead to increased survival in the soil or an increase in symbiotic competency, a desired outcome in areas where nitrogen fertilizer is not readily accessible or a financially feasible input for farmers. In addition to the development of rhizobia that are better suited to various soils and ultimately a better inoculum for legume crops, the results presented here suggest that stress interactions may be important in other contexts and suggests that measuring bacterial stress responses as a coordinated system could be important in understanding other bacteria that interact with specialized environments. The limited availability of nitrogen and phosphate in both terrestrial and aquatic environments suggests that coordination of stress responses may be common.

## Data Availability Statement

The datasets presented in this study can be found in online repositories. The names of the repository/repositories and accession number(s) can be found below: https://www.ncbi.nlm.nih.gov/, PRJNA773132.

## Author Contributions

KH, SY, and MK contributed to conception and design of the study. KH acquired the data and wrote the first draft of the manuscript. KH, JP, and SY analyzed the data. KH, JP, SY, and MK contributed to data interpretation. KH, JP, and MK contributed figures. All authors contributed to manuscript revisions, and read and approved the submitted version.

## Conflict of Interest

The authors declare that the research was conducted in the absence of any commercial or financial relationships that could be construed as a potential conflict of interest.

## Publisher’s Note

All claims expressed in this article are solely those of the authors and do not necessarily represent those of their affiliated organizations, or those of the publisher, the editors and the reviewers. Any product that may be evaluated in this article, or claim that may be made by its manufacturer, is not guaranteed or endorsed by the publisher.

## Footnotes

1
http://www.kazusa.or.jp/codon/cgi-bin/showcodon.cgi?species=266834
