# Supplementary material for: The Sinorhizobium meliloti Nitrogen Stress Response Changes Radically in the Face of Concurrent Phosphate Stress
Source: Front Microbiol. 2022 Jan 27;13:800146. doi: 10.3389/fmicb.2022.800146 (PMC8829014; doi:10.3389/fmicb.2022.800146)
Supplement: Supplementary file 7 [file Data_Sheet_1.PDF]

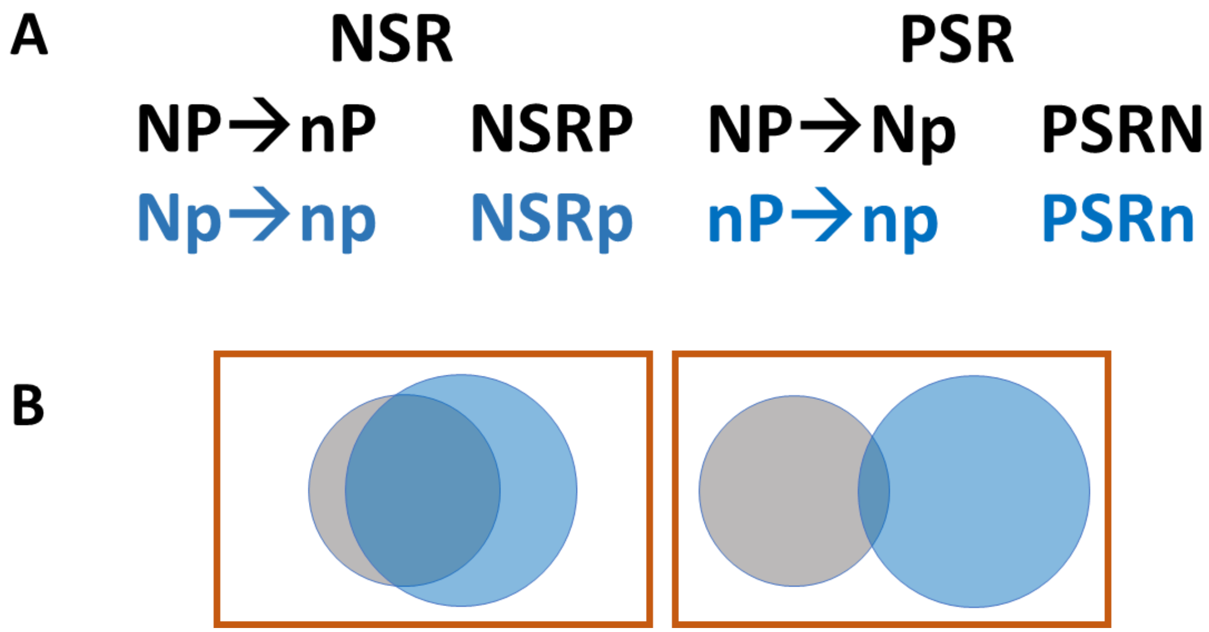

Supplementary Figure S1. Graphic representation of the relationship between nutrient stress responses under different conditions. (A) Stress responses are defined in this paper as the set of genes where expression changed 2-fold between the stress condition and the non-stress condition. Two nitrogen stress responses (NSRs) were measured in this work, the NSR corresponding to limited nitrogen under high phosphate [**NSRP**] and the NSR corresponding to limited nitrogen under low phosphate [**NSRp**]. Similar phosphate stress responses (PSRs) were determined under conditions of high nitrogen [**PSRN**] and low nitrogen [**PSRn**]. **(B)** Possible relationships between stress responses in different contexts. The set of genes that could potentially respond to a stress is indicated by the rectangles. A different number and group of these genes might respond to the “same” stress under different experimental conditions (gray and blue circles) and could be represented by a number of different Venn diagrams similar to the two shown here.
