# Supplementary material for: The Sinorhizobium meliloti Nitrogen Stress Response Changes Radically in the Face of Concurrent Phosphate Stress
Source: Front Microbiol. 2022 Jan 27;13:800146. doi: 10.3389/fmicb.2022.800146 (PMC8829014; doi:10.3389/fmicb.2022.800146)
Supplement: Supplementary file 8 [file Data_Sheet_2.pdf]

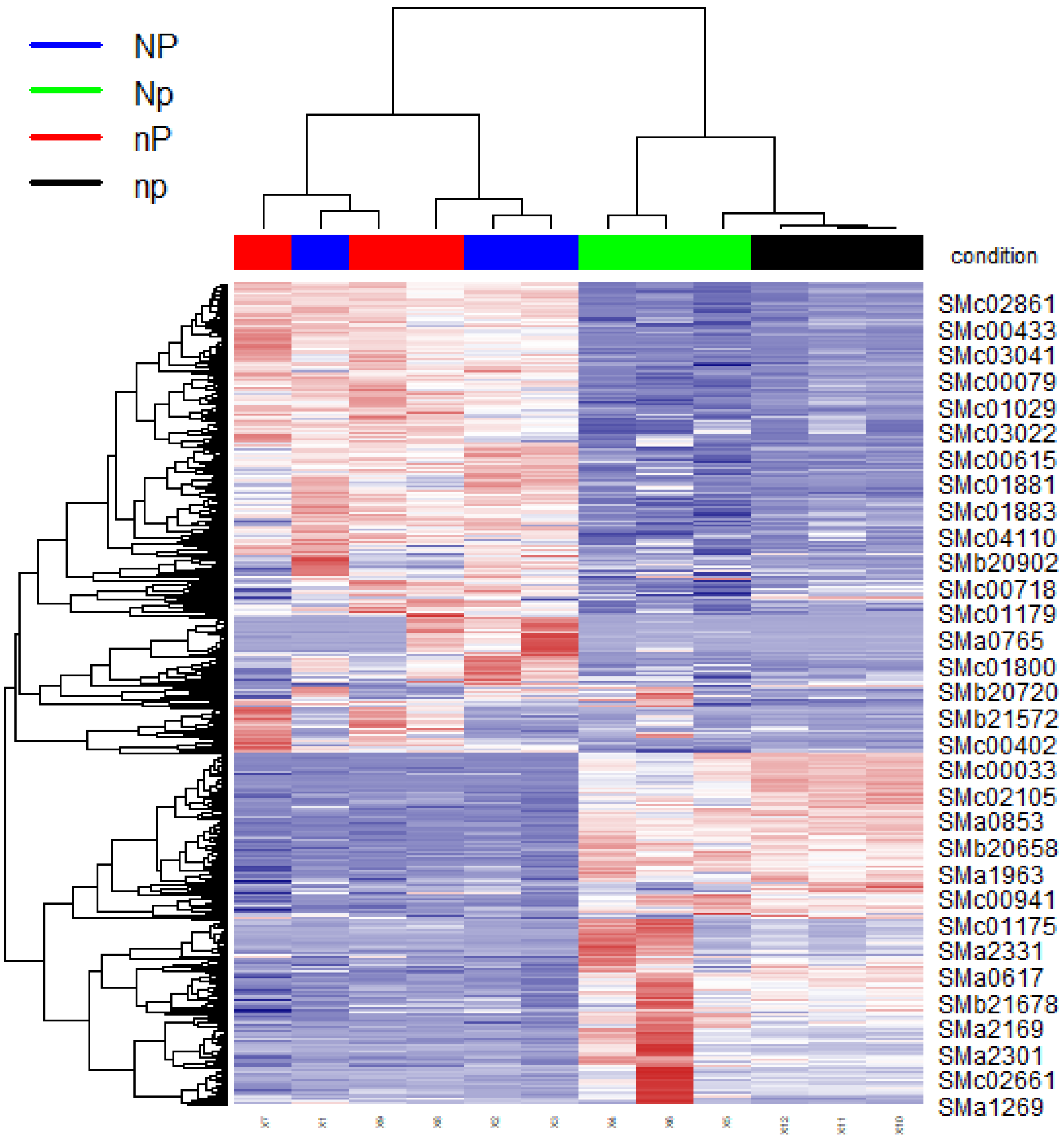

Supplementary Figure S2. Heatmap of significant gene expression changes in 1021 pstC+. A heatmap of the significant differentially expressed genes for each 1021 pstC+ sample. Samples were grown in one of four media conditions as described in the paper and labeled here as NP, Np, nP, or np and color coded along the top row labeled “condition”. Within the heatmap, gene expression is visualized where red indicates increased expression and blue indicates decreased expression relative to other samples in the dataset. The intensity of the red/blue color represents the magnitude of the value. Arbitrarily selected genes are labeled on the right side of the figure.
