# Supplementary material for: The Sinorhizobium meliloti Nitrogen Stress Response Changes Radically in the Face of Concurrent Phosphate Stress
Source: Front Microbiol. 2022 Jan 27;13:800146. doi: 10.3389/fmicb.2022.800146 (PMC8829014; doi:10.3389/fmicb.2022.800146)
Supplement: Supplementary file 9 [file Data_Sheet_3.pdf]

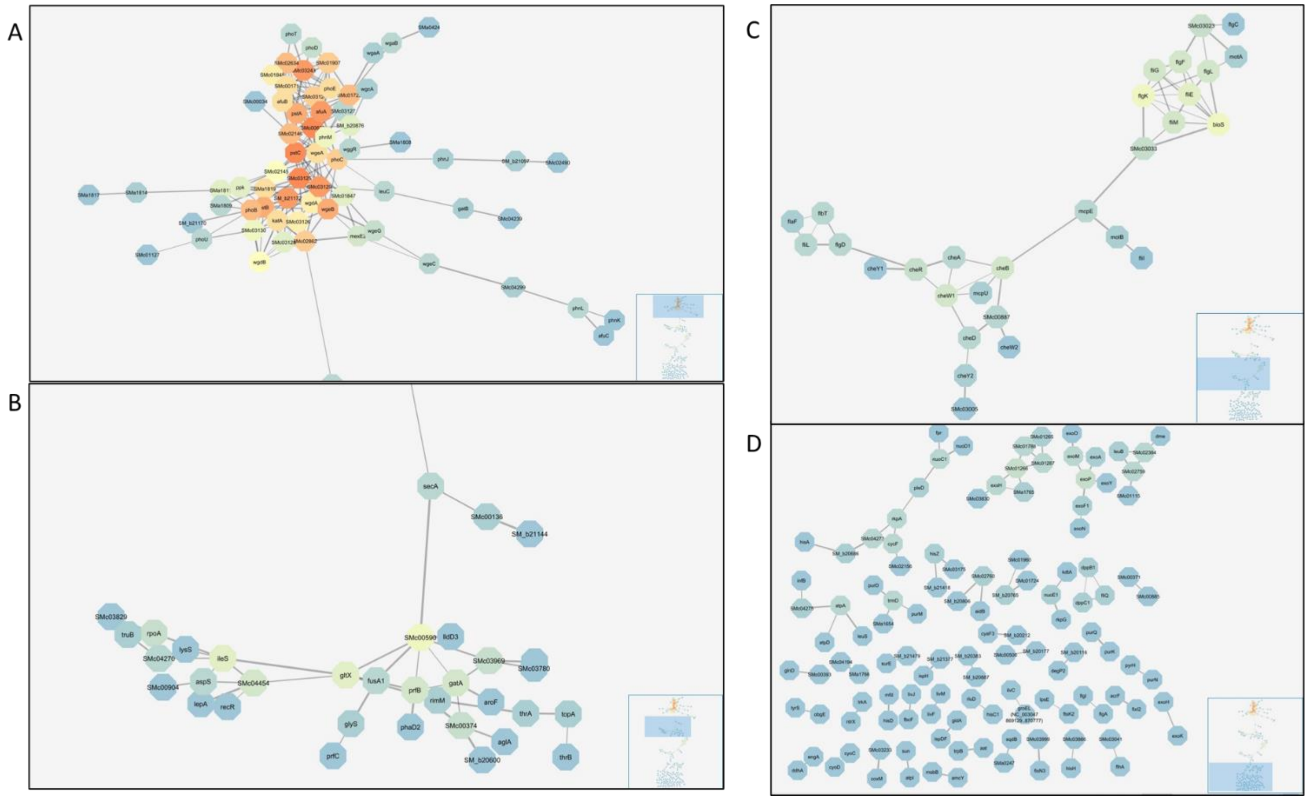

Supplementary Figure S3. Network map of connected genes based on weighted co-expression analysis. Network map created in Cytoscape depicting genes that are linked based on their expression patterns and correlation with each other when evaluated with all 4 media conditions**.** Detailed views of the network map start at the top **(A)** and move downward to the bottom **(D)**. Colors represent degrees and depict how connected a gene is to other genes in the map. The color scheme starts with blue as the least connected (fewest degrees) and flows through green to yellow to orange as the most connected (highest degrees). The bottom right inset in each panel highlights the area of the full network map that is enlarged.
